# Tooth Color Change Following Bleaching with a Desensitizing Double-Layer Technique

**DOI:** 10.3390/bioengineering13080864

**Published:** 2026-07-27

**Authors:** Basil Almutairi, Abdullah Alshehri, Hamad Algamaiah, Carlos A. Jurado, Silvia Rojas-Rueda, Muadh A. AlGomaiah, Abdulrahman Alshabib

**Affiliations:** 1 Department of Restorative Dental Science, College of Dentistry, King Saud University, P.O. Box 60169, Riyadh 11545, Saudi Arabia; 2 Conservative Dental Science Department, College of Dentistry, Prince Sattam Bin Abdulaziz University, Al-Kharj, Riyadh 16245, Saudi Arabia; 3 School of Dental Medicine, Ponce Health Sciences University, Ponce, PR 00732, USA; 4 Department of Pediatric Dentistry and Orthodontics, College of Dentistry, King Saud University, P.O. Box 60169, Riyadh 11545, Saudi Arabia

**Keywords:** tooth bleaching, tooth whitening, potassium nitrate, double-layer technique, color change

## Abstract

This in vitro study evaluated the effect of incorporating a desensitizing layer between the tooth surface and bleaching gel on tooth color change and treatment time during in-office bleaching procedures. The novelty of this study lies in assessing whether a double-layer technique, previously proposed to modify the diffusion behavior of potassium nitrate and hydrogen peroxide, can maintain whitening efficacy while reducing overall application time. Sixty caries-free third molars were randomly assigned to three groups (n = 20). The CTRL group received 25% hydrogen peroxide (HP) for 30 and 45 min; the DL group received a double-layer application consisting of 5% potassium nitrate followed by 25% HP for the same time intervals; and the MIXED group received 3% potassium nitrate incorporated into 40% HP. Tooth color was measured with a digital spectrophotometer at the baseline and after each treatment interval. Repeated measures ANOVA and pairwise *t*-tests were used to evaluate differences among groups and treatment times. No significant differences in color change were found among the groups at either evaluation time. The ΔE values obtained with the double-layer technique at 30 min were not significantly different from those observed in the CTRL and MIXED groups at 45 min (*p* = 0.75 and *p* = 0.33, respectively). Within the limitations of this in vitro study, no significant differences in tooth color were detected between the double-layer technique and conventional bleaching approaches, though sufficient whitening outcomes were achieved in a shorter treatment time. These findings suggest that this technique may represent a clinically relevant strategy to improve the efficiency of in-office bleaching protocols without compromising whitening efficacy.

## 1. Introduction

Recent advancements in biomimetic dentistry, particularly in materials and clinical techniques, have substantially transformed contemporary dental treatment strategies [1]. These innovations aim not only to restore the mechanical properties of natural teeth, such as their strength, elasticity, and biological compatibility, but also to replicate their esthetic characteristics. Biomimetic bleaching, for example, has been associated with smoother enamel surfaces, decreased sensitivity, and reduced plaque accumulation, resulting in both immediate and sustained whitening outcomes [2].

Despite their effectiveness, tooth sensitivity remains one of the most frequently reported adverse effects of whitening procedures, with 20% to 100% of patients experiencing sensitivity following in-office treatments [3]. This sensitivity is largely attributed to the use of high concentrations of hydrogen peroxide. To address this issue, various strategies have been proposed, including the use of desensitizing agents. Potassium nitrate is among the most commonly used agents, acting by diffusing through the enamel and dentin to reduce nerve excitability [4,5,6]. Once potassium ions reach the nerve endings, they interfere with nerve impulse transmission, thereby reducing or eliminating pain.

Different application protocols have been suggested to enhance the effectiveness of desensitizing agents, including their use prior to whitening procedures for periods of 15 to 30 min. While this approach has shown effectiveness in decreasing sensitivity, it introduces additional chairside time. To improve efficiency, manufacturers have incorporated desensitizing agents directly into whitening gels, reducing treatment time; however, this modification does not consistently result in decreased sensitivity [7,8,9,10,11].

Hydrogen peroxide, due to its low molecular weight and rapid oxidative capacity, diffuses through dental tissues more quickly than desensitizing agents, limiting their ability to act effectively [12,13,14]. Consequently, by the time desensitizing agents reach the pulp to exert their effects, hydrogen peroxide may have already penetrated the pulp tissue, caused cellular damage and activated nociceptive receptors, which leads to pain perception.

A novel approach, known as the double-layer technique, involves the sequential application of a potassium nitrate desensitizing gel followed by a whitening gel. This method is designed to allow the earlier diffusion of the slower-acting desensitizing agent toward the pulp, while the hydrogen peroxide must first diffuse through the desensitizing layer before reaching the tooth structure. This configuration has the potential to reduce both treatment time and the sensitivity associated with whitening procedures. However, previous studies have reported that the inclusion of desensitizing agents may compromise whitening efficacy, and there is limited evidence regarding the impact of the double-layer technique on overall color change [5,7,8,9,10,11,12,13,14,15,16,17,18,19,20]. Preliminary investigations have primarily focused on diffusion characteristics to evaluate this technique’s feasibility [12,13,14].

Currently, there is insufficient evidence assessing the effectiveness of this technique compared to conventional whitening methods in terms of total color change. Therefore, the aim of this study is to evaluate the effect of introducing a desensitizing gel layer between the tooth surface and the whitening agent on (1) tooth color change in comparison with standard whitening techniques and on (2) treatment duration. The null hypotheses were that no differences would be observed in tooth color change between the double-layer technique and conventional whitening methods, that no differences would exist between 30 min and 45 min whitening protocols, and that no differences would be found in tooth color change among different whitening techniques at various time points.

The novelty of the present study lies in the evaluation of a double-layer bleaching technique designed to integrate desensitizing and whitening procedures into a single clinical protocol. Although previous investigations have assessed the diffusion behavior of potassium nitrate and hydrogen peroxide, limited evidence is available regarding whether placing a desensitizing layer between the tooth surface and the bleaching gel affects the final whitening outcome. This study therefore provides new information by comparing the color changes produced by the double-layer technique with conventional hydrogen peroxide bleaching and a commercially available bleaching gel containing potassium nitrate. In addition, the study evaluates whether this approach can achieve comparable whitening efficacy in a shorter treatment time, which may be clinically relevant for improving patient comfort and reducing chairside time during in-office bleaching procedures.

## 2. Materials and Methods

The sample size was determined using STATA 16, with an alpha level of 0.05 and 80% power, to detect differences among the null hypotheses. Based on this calculation, the required total sample size for the study was 48 specimens; however, 60 specimens were ultimately included, with 20 samples allocated to each group. Extracted human third molars were used following ethical approval from the institution (REC-HSD-90-2021). The teeth were stored in distilled water at room temperature. They were then thoroughly cleaned with plain pumice (Preppies, Whipmix, Louisville, KY, USA) and prophy cups (Young Dental, Algonquin, IL, USA). The teeth were inspected for caries, restorations, fractures, and cracks, and any teeth presenting these conditions were excluded. The teeth were sectioned 3 mm apical to the CEJ, using a low-speed diamond saw (IsoMet 1000, Buehler, Lake Bluff, IL, USA) under continuous water irrigation to prevent heat generation and minimize sectioning artifacts, and the pulpal tissues were removed. A standardized treatment area measuring 6 mm in diameter was established on the buccal surface using varnish.

The teeth were randomly allocated into three groups (n = 20) and stored individually in sealed glass vials labeled with identification numbers on the side. According to the manufacturer’s instructions, two whitening application times were evaluated:

- **Thirty-minute application:** The whitening agent was applied to all groups for two cycles of 15 min each, for a total of 30 min. The specimens were then rinsed, cleaned, and stored in glass vials for 24 h. Color measurements were recorded after 24 h.
- **Forty-five-minute application:** After the 24 h color measurement, the whitening agent was reapplied to all groups for an additional 15 min, following the initial 30 min treatment, resulting in a total application time of 45 min. The samples were then rinsed, cleaned, and stored in glass vials for 24 h before color measurements were taken.

The specimens were divided into three groups based on the whitening treatment received: Group A (CTRL), Group B (DL), and Group C (MIXED) (Table 1). In accordance with the manufacturer’s instructions, the whitening agents used in the CTRL and DL groups were continuously activated with a light-emitting diode (LED) lamp (Zoom WhiteSpeed, Philips Oral Healthcare, Los Angeles, CA, USA), with a peak wavelength of 466 nm and set at high intensity (190 mW/cm^2^). In the CTRL, DL, and MIXED groups, the whitening gel was replenished every 15 min.

**Figure 1:**
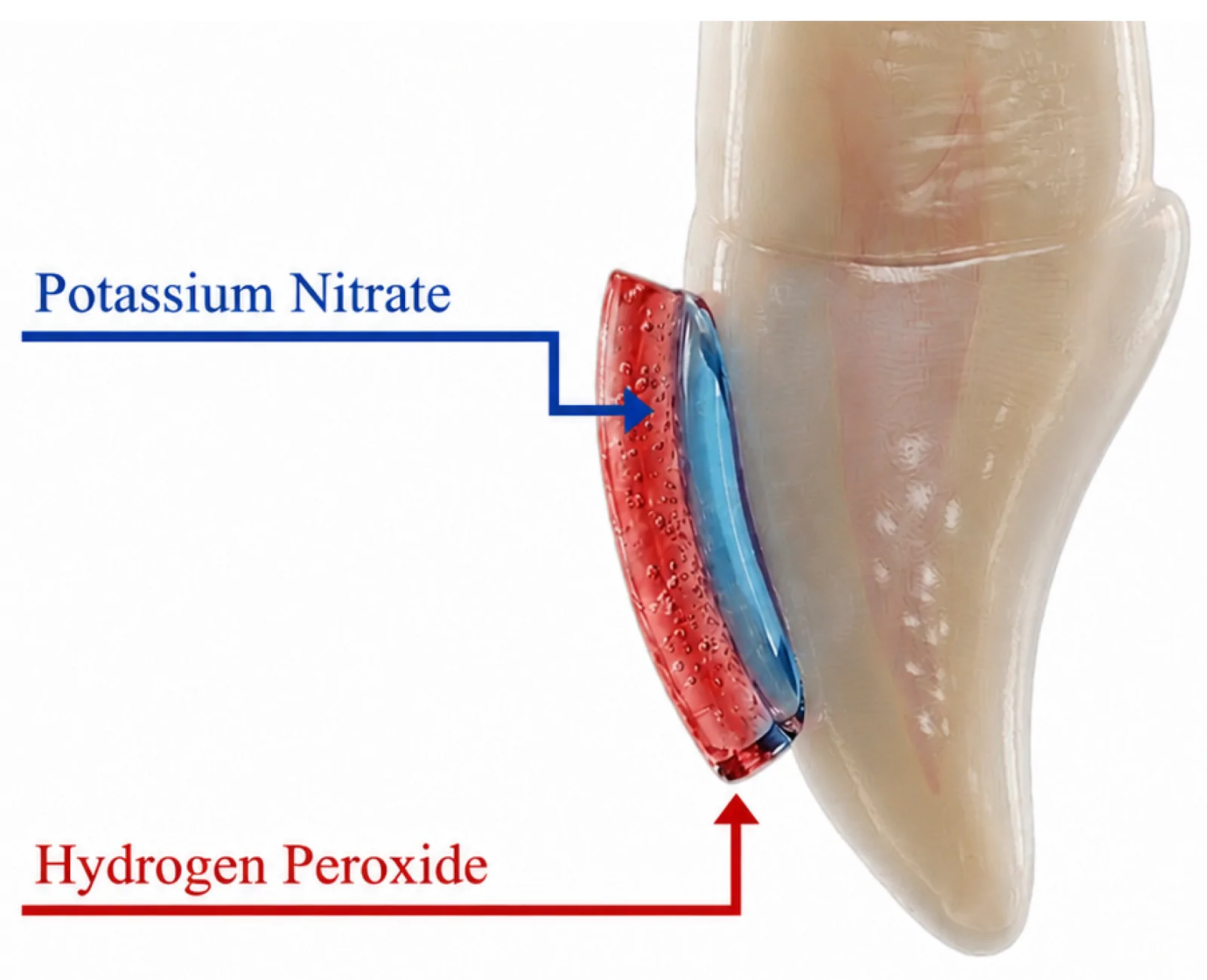
Illustration of the double-layer technique on tooth surface.

Tooth color was assessed at baseline (T0), 24 h after the 30 min treatment (T1), and 24 h after the 45 min treatment (T2). A spectrophotometer (VITA Easyshade Advance 4.0, VITA Zahnfabrik, Bad Säckingen, Germany) was used to measure the color of all specimens at each time point and to obtain the L* (lightness), a* (green–red axis), and b* (blue–yellow axis) values. Prior to each measurement session, the spectrophotometer was calibrated according to the manufacturer’s instructions.

**Figure 2:**
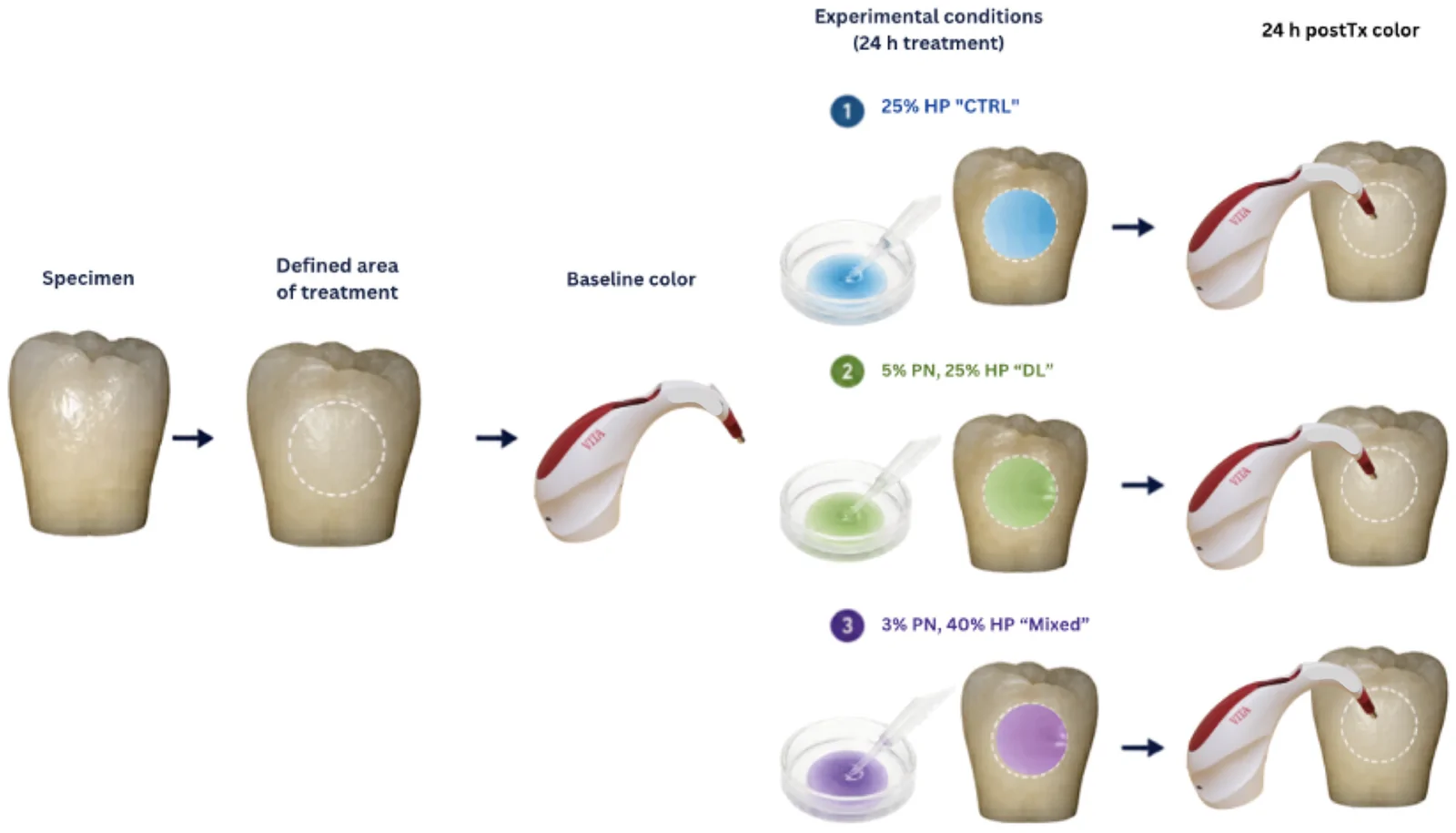
Schematic drawing describing the study design.

Color measurements were obtained with a digital spectrophotometer under standardized conditions using a color-matching box (GTI MiniMatcherR, GTI Graphic Technology, Inc., Newburgh, NY, USA). Three readings were taken for each specimen and the mean value was calculated and used for analysis. The CIELab color coordinates were recorded and the total color change $\left(\Delta E\right)$ was calculated using the following formula: ΔE = (ΔL*^2^ + Δa*^2^ + Δb*^2^)^1/2^ (Figure 2).

Following Shapiro–Wilk and Levene tests that confirmed data normality and the homogeneity in the variance assumptions, a repeated measures ANOVA model was used (α = 0.05). This model allowed for a test of any difference in color change due to method or due to time. Follow-up pairwise *t*-tests were used to further explore any difference due to method or time (α = 0.05).

## 3. Results

### 3.1. Color Change Within Tooth Whitening Group

After tooth-whitening treatment, all three groups demonstrated significant color changes compared with the baseline across the four color change variables (*p* < 0.001 in all cases). The color change values recorded 24 h after the 30 min (T1) and 45 min (T2) treatments are presented in Table 2.

Table 2 Tooth color (ΔL*, Δa*, Δb*, ΔE*) at all time points relative to baseline, including both 30 min (T1) and 45 min (T2) post tooth whitening.

#### 3.1.1. Group Comparison

When overall color change from baseline was analyzed across both time points (T1 and T2), no significant differences were observed between any pair of groups. At T1, the comparisons were as follows: CTRL versus DL, *p* = 0.9483; CTRL versus MIXED, *p* = 0.9981; and DL versus MIXED, *p* = 0.9999. At T2, the comparisons were as follows: CTRL versus DL, *p* = 1.0000; CTRL versus MIXED, *p* = 0.9993; and DL versus MIXED, *p* = 1.0000.

#### 3.1.2. Comparison: 30 Min (T1) Versus 45 Min (T2)

The results showed significant differences in overall color change between Time 1 and Time 2 among the treatment groups, except for the DL technique and the ΔL values in the control group, as shown in Table 2.

#### 3.1.3. Comparison Between DL, Time 1, and CTRL and MIXED, Time 2

To assess the efficacy of the double-layer protocol, separate *t*-tests were performed, comparing it with the 45 min protocols. No significant differences were found in lightness $\left(\Delta L\right)$ $\left(p>0.05\right)$. For chroma shift $\left(\Delta a\right)$, the comparison between DL at Time 1 and CTRL at Time 2 was not significant $\left(p=0.184\right)$, whereas the comparison between DL at Time 1 and MIXED at Time 2 was significant $\left(p=0.007\right)$. No significant differences were found for the blue–yellow change $\left(\Delta b\right)$ $\left(p>0.05\right)$. For overall color change $\left(\Delta E\right)$, the comparison between DL at Time 1 and CTRL at Time 2 was not significant $\left(p=0.75\right)$, and the comparison between DL at Time 1 and MIXED at Time 2 was also not significant $\left(p=0.33\right)$ (Table 3).

## 4. Discussion

This study offers new insights into the clinical applicability of a double-layer bleaching method in which a desensitizing agent and hydrogen peroxide are applied sequentially. Previous research has primarily examined the diffusion characteristics of this technique, while relatively little is known about its ability to preserve bleaching effectiveness while shortening the overall treatment period. Accordingly, the present investigation expands the available evidence by assessing the tooth color change produced by the double-layer protocol and comparing its performance with that of conventional in-office bleaching techniques.

At-home tooth whitening is still regarded as the gold standard because it produces reliable results and enables patients to complete treatment at their convenience. However, the growing preference for faster esthetic improvements and reduced treatment times has increased the appeal of in-office whitening, which can also provide effective clinical outcomes [9]. However, a major disadvantage of in-office procedures is the extended chairside time often needed to complete treatment [10]. Different strategies have been introduced to shorten in-office treatment duration, including increasing the concentration of HP and using light-activated HP gels. Although higher HP concentrations enhance whitening efficacy, they are also associated with adverse effects such as tooth sensitivity and changes in tooth surface morphology [9,14]. Consequently, alternative approaches have been explored. Lower concentrations of HP were demonstrated to be effective whitening outcomes when combined with a light source and when treatment protocols were carefully designed, but complications still persisted [15,16]. The incorporation of desensitizing and remineralizing agents into whitening protocols was later introduced to minimize these drawbacks [17,18]. While these additional agents reduced tooth sensitivity and protected tooth surfaces, they also increased the number of clinical steps, ultimately prolonging treatment time, which was originally one of the primary concerns manufacturers sought to address.

The present study evaluated an innovative double-layer, single-application technique in which a potassium nitrate layer is covered by an external hydrogen peroxide layer. This layered configuration integrates both agents into a single protocol, reducing the overall application time of the desensitizing and whitening procedure. It is also presumed to facilitate potassium nitrate diffusion while temporarily delaying hydrogen peroxide diffusion, since incorporating both agents into a single gel formulation had previously proven ineffective due to the rapid oxidation and diffusion characteristics of HP [9]. This approach demonstrated higher concentrations of potassium nitrate and hydrogen peroxide within the tooth structure compared with other commercially available techniques while requiring less treatment time. Since hydrogen peroxide diffusion is directly associated with tooth color alteration [14], this technique may improve whitening efficiency while simultaneously decreasing overall treatment duration compared with currently available methods.

For group comparison, the experimental protocol used in this study was evaluated against two commercially available whitening systems with a well-established history of successful tooth color enhancement. All groups produced clinically perceptible color changes (ΔE) exceeding the threshold detectable by the naked eye, highlighting the clinical relevance of the findings [19]. These results support the first hypothesis that no significant differences would exist among the groups regarding overall tooth color change. Multiple variables influenced the final color outcome in this study. In the MIXED group, the elevated HP concentration appeared to be the primary contributing factor, as greater HP concentrations have been associated with improved whitening results. The use of 40% HP may produce enhanced whitening while eliminating the need for additional auxiliary devices such as light activation systems [20,21]. However, within the present investigation, differences in HP concentration (25%, 40%) did not significantly affect overall color change, as the CTRL group demonstrated performance comparable to the MIXED group. Moreover, previous investigations have reported superior whitening outcomes with lower HP concentrations compared with higher concentrations [22,23]. In the CTRL group, light activation likely contributed to the effectiveness of the whitening gel.

To reduce the undesirable effects associated with high HP concentrations and light activation [24,25,26], a desensitizing barrier layer was incorporated into the DL group to minimize tooth sensitivity. Because both layers shared the same glycerin-based formulation, the hydrogen peroxide’s efficacy was not compromised, as it was still able to diffuse through the desensitizing layer and interact with chromogenic molecules (the substances that undergo a distinct color change) inside the tooth structure. The ΔE values measured after 24 h in this study differed from those reported in in vivo investigations. This discrepancy may be related to the inherent differences between in vitro and in vivo conditions [27].

The current literature contains limited evidence regarding the use of desensitizing agents as temporary barrier layers and their effect on overall tooth color change. However, previous studies have evaluated color change when desensitizing agents were applied either before or concurrently with bleaching agents. Several desensitizing compounds have been investigated, including potassium nitrate [28] and fluoride [29,30]. These studies found no significant differences in overall color change between protocols that incorporated desensitizing agents and those in which bleaching treatment was performed alone. These findings agree with the results of the present study and suggest that the use of desensitizing agents does not compromise the expected whitening effect.

Hydrogen peroxide diffusion is closely associated with tooth color change because increased diffusion results in the greater availability of free radicals capable of interacting with pigmented molecules [12,13]. The second hypothesis stated that whitening efficacy would not differ among the treatment groups when the two application times, 30 and 45 min, were compared. The findings of the present study led to the rejection of this hypothesis. Previous investigations have demonstrated a direct association between application time and hydrogen peroxide diffusion [31,32], suggesting that longer treatment durations facilitate greater peroxide diffusion and, consequently, enhance the whitening effect. A previous in vitro investigation demonstrated greater HP diffusion through the tooth structure using the DL technique compared with commercially available systems. Additionally, that study reported higher HP diffusion with the DL protocol at 30 min than with commercial systems applied according to the manufacturer’s full recommended treatment time (45 min per session) [14]. In the present investigation, the whitening efficacy of the DL technique was evaluated at 30 min and compared with the CTRL and MIXED groups, applied for the manufacturer-recommended duration of 45 min. No statistically significant differences were observed among the groups regarding overall color change, indicating that the DL protocol achieved a performance comparable to those achieved with the CTRL and MIXED protocols.

Although this in vitro study demonstrated promising results, its controlled laboratory design primarily evaluates product efficacy rather than clinical effectiveness. Therefore, future in vivo clinical investigations are necessary to assess this technique under real clinical conditions and to determine the influence of variables such as patient compliance, dietary habits, beverage consumption, and the overall impact of the technique on tooth color change.

## 5. Conclusions

Under the limitations of this study, no significant differences in tooth color were detected between the double-layer technique and conventional bleaching approaches. The use of potassium nitrate as a barrier layer between hydrogen peroxide and the tooth structure did not negatively influence overall tooth color change, as the comparison between DL at Time 1 and CTRL at Time 2 was not significant. Additionally, no significant differences were detected between the total color change under the double-layer protocol after 30 min and the total color change produced by commercial techniques applied for 45 min.

## Figures and Tables

**Table 1:** Tooth-whitening treatment groups and materials used.

Group	Treatment
Group A (CTRL)	25% hydrogen peroxide (Zoom Chairside Whitening Gel, Philips Oral Healthcare, Los Angeles, CA, USA)
Group B (DL)	Double-layer treatment consisting of 5% potassium nitrate (Relief ACP, Philips Oral Healthcare, Los Angeles, CA, USA) and 25% hydrogen peroxide (Zoom WhiteSpeed, Philips) (Figure 1).
Group C (MIXED)	Gel containing 3% potassium nitrate incorporated into 40% hydrogen peroxide (Opalescence Boost PF, Ultradent, South Jordan, UT, USA)

**Table 2:** Tooth colour changes (mean ± SD) at two time points for the three study groups.

Tooth Colour Parameter (Time Point)	CTRL (n = 30)	DL (n = 30)	MIXED (n = 30)	Overall *p* Value *	Post Hoc Comparisons (Bonferroni)
	Mean ± SD	Mean ± SD	Mean ± SD		
Time point 1 (T1; 30 min)					
ΔL*	2.3 ± 4.9 ^a^	4.0 ± 3.6 ^b^	1.3 ± 5.9 ^a^	<0.001	DL > CTRL (*p* = 0.43) DL > MIXED (*p* < 0.001) CTRL = MIXED (*p* = 1.000)
Δa*	−1.2 ± 2.1 ^a^	−0.9 ± 1.5 ^ab^	−1.8 ± 1.3 ^b^	<0.001	CTRL > MIXED (*p* < 0.001) DL = (CTRL + MIXED) (*p* = 0.099)
Δb*	−5.2 ± 2.9 ^a^	−6.2 ± 5.0 ^b^	−4.1 ± 3.7 ^c^	<0.001	DL < CTRL < MIXED (all *p* < 0.05)
ΔE*_ab_ (CIE76)	6.7 ± 4.9 ^a^	8.6 ± 4.6 ^b^	7.9 ± 2.9 ^b^	<0.001	DL > CTRL (*p* < 0.01) MIXED > CTRL (*p* < 0.05) DL = MIXED (*p* = 0.45)
**Time point 2 (T2; 45 min)**					
ΔL*	4.8 ± 4.2 ^a^	5.2 ± 3.9 ^a^	4.7 ± 6.2 ^a^	<0.001	No significant pairwise differences
Δa*	−2.4 ± 2.0 ^a^	−1.6 ± 1.6 ^b^	−2.9 ± 1.5 ^c^	<0.001	DL > CTRL (*p* < 0.001) DL > MIXED (*p* < 0.001) CTRL > MIXED (*p* < 0.05)
Δb*	−9.5 ± 3.6 ^a^	−9.3 ± 5.7 ^a^	−8.0 ± 6.1 ^b^	<0.001	CTRL = DL (*p* = 1.000) Both < MIXED (*p* < 0.05)
ΔE*_ab_ (CIE76)	11.3 ± 5.2 ^a^	11.9 ± 5.0 ^a^	12.3 ± 4.6 ^a^	<0.001	No significant pairwise differences

Values are presented as mean ± standard deviation (SD). ΔL*: Lightness (whiteness); Δa*: Red–green axis; Δb*: Yellow–blue axis; ΔE*_ab_ (CIE76): Overall colour difference calculated using the CIE76 formula: ΔE*_ab_ = √[(ΔL*)^2^ + (Δa*)^2^ + (Δb*)^2^]. * One-way ANOVA across the three groups. When the overall *p* value was < 0.05, pairwise comparisons were performed using the Bonferroni post hoc test. Different superscript letters within the same row indicate statistically significant differences (*p* < 0.05). Groups sharing the same letter are not significantly different.

**Table 3:** Associated significance probability values of color parameters between different time points.

Color Change		Associated Significance Probability	
Time Interval	Dimension	T2 CTRL Group (45 Min)	T2 MIXED Group (45 Min)
**T1 DL group** **(30 min)**	ΔL	1	1
	Δa	0.184	0.007
	Δb	1	1
	ΔE	0.75	0.33

## Data Availability

Data presented in this study are available on request from the corresponding authors.

## Abbreviations

The following abbreviations are used in this manuscript:

HP	hydrogen peroxide
DL	double layer
CTRL	control group
MIXED	potassium nitrate incorporated into hydrogen peroxide
LED	light-emitting diode
ΔE	total color change
ΔL*	change in lightness
Δa*	change in red–green coordinate
Δb*	change in yellow–blue coordinate
CIELab	Commission Internationale de l’Éclairage L*a*b* color system
T0	baseline measurement
T1	measurement after 30 min treatment
T2	measurement after 45 min treatment
SD	standard deviation
ANOVA	analysis of variance
ACP	amorphous calcium phosphate
